# Supplementary material for: Kindled emotions: Commemoration and the importance of meaning making, support and recognition
Source: PLoS One. 2023 Apr 24;18(4):e0284763. doi: 10.1371/journal.pone.0284763 (PMC10124837; doi:10.1371/journal.pone.0284763)
Supplement: S1 Appendix — (DOCX) [file pone.0284763.s003.docx]

**Supporting Information**

**S1 Appendix: Factor analysis**

A principal axis factor analysis was conducted on the 10 items with oblique rotation (direct oblimin). The Kaiser-Meyer-Olkin measure verified the sampling adequacy for the analysis, KMO = .82 (‘meritorious’ according to Hutcheson & Sofroniou [1]), and all KMO values for individual items were greater than .70, which is well above the acceptable limit of .5 [2]. An initial analysis was run to obtain eigenvalues for each factor in the data. Two factors had eigenvalues over Kaiser’s criterion of 1 and in combination explained 49.10% of the variance. Table A1 shows the factor loadings after rotation. The items that cluster on the same factor suggest that factor 1 represents negative emotions and factor 2 represents positive emotions.

**S1 Table. Summary of exploratory factor analysis results for emotions (*n* = 290).**

|  | Rotated factor loadings | |
| --- | --- | --- |
| Item | **Negative emotions** | **Positive emotions** |
| Sad | **.75** | -.01 |
| Downhearted | **.76** | -.02 |
| Angry | **.78** | -.05 |
| Afraid | **.73** | -.04 |
| Ashamed | **.64** | .14 |
| Inspired | .15 | **.59** |
| Happy | -.29 | **.71** |
| Proud | .14 | **.61** |
| Concentrating | -.12 | **.60** |
| Calm | -.40 | **.47** |
| Eigenvalues | 3.37 | 1.54 |
| % of variance | 33.71 | 15.38 |
| α | .86 | .74 |

Note: Factor loadings > .40 appear in bold

**References**1. Hutcheson G, Sofroniou N. The multivariate social scientist. London: Sage; 1999.

2. Field A. Discovering statistics using IBM SPSS statistics: And sex and drugs and Rock “N” Roll. 4 ed. London: Sage; 2013.
