# Supplementary material for: Kindled emotions: Commemoration and the importance of meaning making, support and recognition
Source: PLoS One. 2023 Apr 24;18(4):e0284763. doi: 10.1371/journal.pone.0284763 (PMC10124837; doi:10.1371/journal.pone.0284763)
Supplement: S2 Appendix — (DOCX) [file pone.0284763.s004.docx]

**Supporting information**

**S2 Appendix: Regression analyses**

**S2.1 Table. Regression 1; Individual characteristics predicting negative emotions at T1 (*n* = 301).**

|  |  |  |  |  |  | 95% CI | |
| --- | --- | --- | --- | --- | --- | --- | --- |
| Predictor | ***R^2^_adj_*** | **b** | **SE** | ***β*** | ***p*** | **LL** | **UL** |
| Model 1 | 0.32*** |  |  |  |  |  |  |
| T0 negative emotions |  | 0.64 | 0.05 | .57 | <.001 | 0.53 | 0.74 |
| Model 2 | 0.34*** |  |  |  |  |  |  |
| T0 negative emotions |  | 0.68 | 0.06 | .60 | <.001 | 0.57 | 0.79 |
| Age |  | 0.75 | 0.31 | .17 | .02 | 0.14 | 1.36 |
| Gender (1=female) |  | 26.18 | 10.72 | .13 | .02 | 5.08 | 47.29 |
| Migration background (1=yes) |  | 6.72 | 11.63 | .03 | .56 | -16.16 | 29.60 |
| War experience (1=no) |  | 32.08 | 15.62 | .15 | .04 | 1.33 | 62.83 |

*** *p* < .001

**S2.2 Table. Regression 2; Individual characteristics predicting positive emotions at T1 (*n* = 287).**

|  |  |  |  |  |  | 95% CI | |
| --- | --- | --- | --- | --- | --- | --- | --- |
| Predictor | ***R^2^ _adj_*** | **b** | **SE** | ***β*** | ***p*** | **LL** | **UL** |
| Model 1 | 0.27*** |  |  |  |  |  |  |
| T0 positive emotions |  | 0.49 | 0.05 | .52 | <.001 | 0.40 | 0.59 |
| Model 2 | 0.29*** |  |  |  |  |  |  |
| T0 positive emotions |  | 0.47 | 0.05 | .50 | <.001 | 0.37 | 0.56 |
| Age |  | 0.02 | 0.26 | .01 | .94 | -0.50 | 0.53 |
| Gender (1=female) |  | -22.92 | 9.21 | -.14 | .01 | -41.05 | -4.79 |
| Migration background (1=yes) |  | -1.20 | 9.80 | -.01 | .90 | -20.49 | 18.09 |
| War experience (1=no) |  | 26.83 | 12.63 | .16 | .03 | 1.98 | 51.69 |

*** *p* < .001

**S2.3 Table. Regression 3; Mental health symptoms predicting negative emotions at T1 (*n* = 296).**

|  |  |  |  |  |  | 95% CI | |
| --- | --- | --- | --- | --- | --- | --- | --- |
| Predictor | ***R^2^ _adj_*** | **b** | **SE** | ***β*** | ***p*** | **LL** | **UL** |
| Model 1 | 0.33*** |  |  |  |  |  |  |
| T0 negative emotions |  | 0.64 | 0.05 | .57 | <.001 | 0.53 | 0.74 |
| Model 2 | 0.34*** |  |  |  |  |  |  |
| T0 negative emotions |  | 0.54 | 0.06 | .48 | <.001 | 0.42 | 0.66 |
| PTS symptoms |  | 1.31 | 0.44 | .18 | .003 | 0.45 | 2.18 |
| Grief reactions |  | -0.40 | 0.76 | -.03 | .60 | -1.90 | 1.10 |

*** *p* < .001

**S2.4 Table. Regression 4; Mental health symptoms predicting positive emotions at T1 (*n* = 282).**

|  |  |  |  |  |  | 95% CI | |
| --- | --- | --- | --- | --- | --- | --- | --- |
| Predictor | ***R^2^ _adj_*** | **b** | **SE** | ***β*** | ***p*** | **LL** | **UL** |
| Model 1 | 0.28*** |  |  |  |  |  |  |
| T0 positive emotions |  | 0.50 | 0.05 | .53 | <.001 | 0.41 | 0.59 |
| Model 2 | 0.27*** |  |  |  |  |  |  |
| T0 positive emotions |  | 0.49 | 0.05 | .52 | <.001 | 0.40 | 0.59 |
| PTS symptoms |  | -0.20 | 0.33 | -.04 | .55 | -0.86 | 0.45 |
| Grief reactions |  | -0.04 | 0.63 | .00 | .95 | -1.29 | 1.20 |

*** *p* < .001

**S2.5 Table. Regression 5; Psychosocial factors predicting negative emotions at T1 (*n* = 293).**

|  |  |  |  |  |  | 95% CI | |
| --- | --- | --- | --- | --- | --- | --- | --- |
| Predictor | ***R^2^ _adj_*** | **b** | **SE** | ***β*** | ***p*** | **LL** | **UL** |
| Model 1 | 0.32*** |  |  |  |  |  |  |
| T0 negative emotions |  | 0.63 | 0.05 | .57 | <.001 | 0.52 | 0.73 |
| Model 2 |  |  |  |  |  |  |  |
| T0 negative emotions | 0.38*** | 0.57 | 0.06 | .51 | <.001 | 0.46 | 0.68 |
| Recognition |  | -0.45 | 3.08 | -.01 | .88 | -6.51 | 5.61 |
| Support |  | -0.68 | 2.75 | -.01 | .81 | -6.09 | 4.73 |
| Meaning making |  | -5.10 | 2.80 | -.10 | .07 | -10.61 | 0.41 |
| Expression |  | 16.33 | 3.38 | .30 | <.001 | 9.68 | 22.98 |
| Personal memories |  | 0.42 | 2.40 | .01 | .86 | -4.31 | 5.14 |

*** *p* < .001

**S2.6 Table. Regression 6; Psychosocial factors predicting positive emotions at T1 (*n* = 279).**

|  |  |  |  |  |  | 95% CI | |
| --- | --- | --- | --- | --- | --- | --- | --- |
| Predictor | ***R^2^ _adj_*** | **b** | **SE** | ***β*** | ***p*** | **LL** | **UL** |
| Model 1 | 0.30*** |  |  |  |  |  |  |
| T0 positive emotions |  | 0.52 | 0.05 | .55 | <.001 | 0.43 | 0.61 |
| Model 2 |  |  |  |  |  |  |  |
| T0 positive emotions | 0.39*** | 0.43 | 0.05 | .46 | <.001 | 0.34 | 0.52 |
| Recognition |  | 5.66 | 2.41 | .13 | .02 | 0.91 | 10.41 |
| Support |  | 5.82 | 2.26 | .15 | .01 | 1.37 | 10.27 |
| Meaning making |  | 7.14 | 2.30 | .18 | .002 | 2.60 | 11.67 |
| Expression |  | -3.33 | 2.67 | -.08 | .21 | -8.59 | 1.93 |
| Personal memories |  | 0.71 | 1.90 | .02 | .71 | -3.03 | 4.45 |

*** *p* < .001

**S2.7 Table. Regression 7; Significant variables from the individual characteristics, mental health symptoms and psychosocial factors predicting** **negative emotions at T1 (*n* = 293).**

|  |  |  |  |  |  | 95% CI | |
| --- | --- | --- | --- | --- | --- | --- | --- |
| Predictor | ***R^2^ _adj_*** | **b** | **SE** | ***β*** | ***p*** | **LL** | **UL** |
| Model 1 | 0.32*** |  |  |  |  |  |  |
| T0 negative emotions |  | 0.63 | 0.05 | .56 | <.001 | 0.52 | 0.73 |
| Model 2 | 0.44*** |  |  |  |  |  |  |
| T0 negative emotions |  | 0.53 | 0.06 | .48 | <.001 | 0.42 | 0.65 |
| Age |  | 0.80 | 0.28 | .18 | .005 | 0.25 | 1.36 |
| Gender (1=female) |  | 25.62 | 9.88 | .12 | .01 | 6.17 | 45.07 |
| War experience (1=no) |  | 69.04 | 15.28 | .33 | <.001 | 38.96 | 99.12 |
| PTS symptoms |  | 2.01 | 0.44 | .28 | <.001 | 1.15 | 2.87 |
| Expression |  | 14.87 | 2.47 | .27 | <.001 | 10.00 | 19.74 |

*** *p* < .001

**S2.8 Table. Regression 8; Significant variables from the individual characteristics, mental health symptoms and psychosocial factors predicting positive emotions at T1 (*n* = 278).**

|  |  |  |  |  |  | 95% CI | |
| --- | --- | --- | --- | --- | --- | --- | --- |
| Predictor | ***R^2^ _adj_*** | **b** | **SE** | ***β*** | ***p*** | **LL** | **UL** |
| Model 1 | 0.30*** |  |  |  |  |  |  |
| T0 positive emotions |  | 0.52 | 0.05 | .55 | <.001 | 0.43 | 0.61 |
| Model 2 | 0.41*** |  |  |  |  |  |  |
| T0 positive emotions |  | 0.41 | 0.05 | .43 | <.001 | 0.32 | 0.50 |
| Gender (1=female) |  | -22.42 | 8.48 | -.14 | .01 | -39.11 | -5.73 |
| War experience (1=no) |  | 20.45 | 8.57 | .12 | .02 | 3.57 | 37.33 |
| Recognition |  | 4.26 | 2.47 | .10 | .09 | -0.60 | 9.13 |
| Support |  | 6.44 | 2.16 | .17 | .003 | 2.18 | 10.70 |
| Meaning-making |  | 5.79 | 2.25 | .14 | .01 | 1.36 | 10.23 |

*** *p* < .001
