# Supplementary material for: Kindled emotions: Commemoration and the importance of meaning making, support and recognition
Source: PLoS One. 2023 Apr 24;18(4):e0284763. doi: 10.1371/journal.pone.0284763 (PMC10124837; doi:10.1371/journal.pone.0284763)
Supplement: Attachment — Submitted filename: Rebuttal letter_2.docx [file pone.0284763.s006.docx]

Dr. A.M. Kamperman
Editor of PLOS ONE

Diemen, The Netherlands, March 28, 2023

Dear Dr. Kamperman,

We hereby submit a revised version of our paper “***Kindled emotions: Commemoration and the importance of meaning making, support and recognition***” for publication in PLOS ONE. We thank the reviewer for her positive feedback and we have made the changes that were suggested:

On page 5, we have corrected the grammatical error as suggested (“the British public was ashamed”). On page 27, we have changed the first sentence as suggested by the reviewer: “Findings in relation to our second aim revealed that only some of the individual characteristics were related to the emotional response after watching the commemoration.”

Furthermore, we have reviewed our reference list to ensure that it is complete and correct. Some small changes, all related to reference style, were made (see Revised Manuscript with track changes).

We hope that the paper is now ready for publication. We look forward to receiving your response.

Kind regards, also on behalf of the other authors,

H.B. Mitima - Verloop

ARQ National Psychotrauma Centre, Diemen, The Netherlands
